# Impact of Hispanic Ethnic Concentration and Socioeconomic Status on Obesity Prevalence in Texas Countie

**DOI:** 10.3390/ijerph9041201

**Published:** 2012-04-11

**Authors:** Jennifer J. Salinas, Elizabeth Rocha, Bassent E. Abdelbary, Jennifer Gay, Ken Sexton

**Affiliations:** 1 Division of Epidemiology, Human Genetics and Environmental Sciences, University of Texas Houston Health Science Center, School of Public Health, Brownsville Regional Campus, Brownsville, TX 78520, USA; Email: Elizabeth.a.rocha@uth.tmc.edu (E.R.); Bassent.e.abdelbary@uth.tmc.edu (B.E.A.); Ken.sexton@uth.tmc.edu (K.S.); 2 Health Promotion and Behavior, College of Public Health, University of Georgia, Athens, GA 30602, USA; Email: jlgay@uga.edu

**Keywords:** Hispanic, education, poverty, obesity, county

## Abstract

The purpose of this study is to determine whether Hispanic ethnic concentration is associated with a higher prevalence of obesity and, if this relationship exists, whether it is affected by the socioeconomic environment. The study uses the Texas Behavioral Risk Factor Surveillance System (BRFSS) linked to 2000 census data to access the relationship between prevalence of obesity, Hispanic ethnic concentration, poverty and level of education at a county-level. The findings suggest that the association of Hispanic ethnic concentration and obesity varies by socioeconomic environment. Although little influence was observed for % poverty, the relationship between Hispanic ethnic concentration and obesity differed by county-level educational attainment. High proportion of residents with a bachelor’s degree is associated with a low prevalence of obesity; counties with both high % Hispanic and high % with Bachelor’s degrees had the lowest prevalence of obesity. Our results suggest that promoting and improving education, perhaps including training on healthful living, may serve as an effective means of curbing current obesity trends and associated health problems in Hispanic and possibly other ethnic communities.

## 1. Introduction

Neighborhood environments that place residents at greater risk for obesity are known as obesogenic environments, and typically lack adequate opportunities for physical activity and have insufficient access to healthy foods [1]. Socioeconomically disadvantaged communities are often obesogenic as evidenced by poor access to fresh fruits and vegetables [2], scarcity of recreational venues (e.g., parks, walking trails, swimming pools), and unsafe conditions and situations (e.g., crime, heavy traffic, gang activity, unleashed dogs) [3]. In addition, residents of poor communities are more likely than residents of more affluent communities to lack access to health care, have inadequate knowledge about healthy lifestyles, reside in dilapidated housing and, in general, to have more stressful and less healthful life [4]. Although there is substantial scientific evidence that the community environment is a risk factor for obesity and associated diseases in certain ethnic groups, particularly African Americans [5,6], a similar causal relationship has not been demonstrated in Hispanic or Mexican American communities.

Research indicates that obesity and related adverse outcomes may be offset by ethnic environment through the salubrious effects of culture [7], healthier ethnic food outlets [8] and closer social ties with fellow residents in the community [9]. Research on Hispanic communities provides potential support for the beneficial effect of ethnic concentration on specific diseases and cause-specific mortality [10,11,12,13]. However, evidence suggests that higher Hispanic ethnic concentration is also associated with a higher prevalence of obesity, which is a well-documented risk factor for several chronic diseases in Mexican Americans [14]. It is possible, therefore, that the health consequences of Hispanic ethnic concentration may be cause-specific and/or multi-factorial. Moreover, the relationship between health and ethnic concentration may depend on variations in socioeconomic conditions of communities [5].

Favorable health outcomes documented in Hispanics by multiple studies are unexpected given this population’s overrepresentation in poverty [15] and there is limited information on the mechanisms by which Hispanic ethnic concentration and community socioeconomic environment influence obesity prevalence. Speculation on causal mechanisms has focused primarily on the cultural environment and access to goods and services [7], but the potential effects of other characteristics of ethnic communities that might also be influential have been largely overlooked.

International studies suggest there is a bifurcation in the relationship between community socioeconomic environment and obesity that depends on the overall economic status of the country [16]. In low- or middle-income countries, socioeconomic environment is positively associated with obesity, whereas in high-income countries socioeconomic environment is negatively associated with obesity [17]. In the United States, greater socioeconomic status is generally negatively associated with obesity, and while variation by ethnicity has been investigated [18] the community context has not been fully explored. It is plausible to postulate that the relationship between Hispanic ethnic concentration and obesity varies with socioeconomic characteristics of the community environment.

This study uses the Texas Behavioral Risk Factor Surveillance System [19] linked to 2000 census data to determine the relationship between county-level prevalence of obesity, Hispanic ethnic concentration, and socioeconomic indicators. Previous studies that have investigated the relationship between community environment and obesity have primarily focused on individual measured outcomes and therefore are only able to make inference at an individual level. Few if any have looked at risk factors for obesity at a population level. This analysis will contribute to the overall knowledge of socioeconomic risk factors for obesity by identify populations that may be as risk, thereby providing direction for public health policy on obesity and interventions at a community-wide level. Community-level interventions are becoming increasingly recognized as effective approaches to reducing obesity For example, policy, system and environmental (PSE) interventions are able to reach a larger population and therefore have greater impact on reducing the health burden of obesity [20]. Moreover, the Centers for Disease Control (CDC) has launched the Communities Putting Prevention to Work (CPPW), which is a major program that provides grants to communities to prevent and reduce obesity using the PSE approach [21]. Therefore, our aims to determine whether Hispanic ethnic concentration is associated with a higher prevalence of obesity and, if this relationship exists, whether it is affected by the socioeconomic environment, provides insight into which type of communities may be more at risk for obesity. We hypothesize that low-income; low-education communities with higher Hispanic ethnic concentrations will have a higher prevalence of obesity than low-income, low-education communities with lower Hispanic ethnic concentration. A finding that would indicate obesity rates in Hispanic communities are affected by differential socioeconomic conditions in the local environment.

## 2. Methods

### 2.1. Procedure

The obesity data used in this paper were gathered from the Texas BRFSS adult statewide survey. The survey asked the participant, “About how much do you weigh without your shoes?” and “About how tall are you without your shoes?” The response to these two questions was used to calculate the Body Mass Index (BMI). An average county-level BMI from the time period between 2000 to 2009 provide by the State of Texas to the research team. Only counties with a sample size of ≥125 were included in the analysis. A BMI score of 30 or above was considered obese. Our sample includes information from 91 of 254 Texas counties, including seven of the 14 counties that border Mexico. The decision to use only the 91 counties was based on county population, with large counties having larger samples. Consequently, the 91 counties in this analysis represent the most populated counties in the state.

Sociodemographic characteristics from the 2000 U.S. Census were linked to the obesity data for 91 Texas counties. Sociodemographic variables included median age, percent poverty, percent Hispanic, and percent graduated with a bachelor’s degree or higher within each corresponding county [22]. Initial evaluation of the normalcy of the distribution was conducted for each variable. Due to significant skewness in the percent with a bachelor’s degree and percent poverty, these two variables were converted into categorical variables based on quartiles. Quartiles were chosen in lieu of a transformation for the regression analysis in order to make results easier to interpret, keeping in mind that the relationship between socioeconomic variables and health outcomes are often non-linear.

Additionally, for the bivariate analysis, categories were created to evaluate the relationship between Hispanic ethnic concentration, poverty and percent with a bachelor’s degree. A total of eight combinations were created: High Hispanic/High % with bachelor’s degree, High Hispanic/Low % with bachelor’s degree, Low Hispanic/High % with bachelor’s degree, Low Hispanic/Low % with bachelor’s degree, High Hispanic/High % poverty, High Hispanic/Low % poverty, Low Hispanic/High % poverty, and Low Hispanic/Low % poverty. Cut points were created based using the 50 percentile distribution for each variable. Those at or above the 50th percentile were classified as “high” and those below were classified as “low”. For example the 50th percentile for % Hispanic is 25.1%, and a county that was 45% Hispanic would be classified as “high”.

### 2.2. Statistical Analysis

Testing was done to determine the stability and reliability of the OLS regression analysis. A Cook-Weisburg test was conducted to determine the presence of heteroskedasticity of % Hispanic on the obesity outcome. The test produced a Chi-square value of 1.54 with a *p* value of 0.2147. Cook’s D was conducted to evaluate for outlier influence in an OLS regression model. Because significant outlier effects were determined, we used a robust regression analysis that corrects for the influence of outliers. A total of 7 models were applied using “rreg” in STATA 11 SE [23].

The ArcGIS [24] mapping program was used to generate maps to demonstrate the distribution of obesity by county in the state of Texas. Layers were produced beginning with a shape file from the Texas Demographer website, which provided a 2000 county map of Texas [25]. The information gathered from the U.S. Census Bureau for each county was joined into the existing 2000 county shape file. Individual maps were completed for each category with the percent obesity displayed in quartiles. An “all attributes map” was then generated using all the layers. 

In order to disentangle the relationship between Hispanic ethnic concentration we preformed a path analysis to determine to what extent related socioeconomic county attributes may contribute to the level of obesity in a Texas county. We make a comparison between % Hispanic and % immigrant and conducted analysis to determine the influence of % immigrant on % Hispanics.

## 3. Results

Table 1 presents descriptive statistics for the 91 Texas counties included in this analysis. On average, nearly one-third of the county population is obese and the average county-level median age is 34.4. While the average county-level proportion living at or below the poverty line is 15.6%, the range is 4.7 to 50.9%. Similarly the average percentage of the county with a bachelor’s degree or more is 18.5%, with a range of 6.97 to 47.3%. Finally, the mean percentage Hispanic is 25.1%, with a range of 1.73 to 97.5%.

Table 2 presents mean % obese by quartiles for the eight combinations of high/low % Hispanic and % with bachelor’s degree or higher and % Hispanic with % at or below the poverty line. Means and *p* values from ANOVA tests are presented for 4 combination categories—*i.e.*, High % Hispanic/High % Bachelor’s Degree, High % Hispanic/Low % Bachelor’s Degree, *etc*. The average county prevalence of obesity was significantly higher in counties that were High % Hispanic/Low % Bachelor’s Degree. The lowest average county prevalence of obesity was in counties that were High % Hispanic and High % Bachelor’s Degree. In terms of poverty, the highest average county obesity prevalence was in counties that were High % Hispanic/ High % Poverty and Low % Hispanic/High % Poverty. These two categories were not statistically different from each other. The lowest average county obesity prevalence was in counties that were High % Hispanic/Low % Poverty.

**Table 1:** Descriptive Analysis on 91 Counties in Texas.

Category	Range	Mean	Standard Deviation
Percent Obesity	16.91–44.2	27.95	5.2
Median Age	23.6–46.3	34.4	3.97
Percent Poverty	4.7–50.9	15.6	7.0
Percent with Bachelor’s or More	6.97–47.3	18.5	7.9
Percent Hispanic	1.73–97.5	25.1	22.9

**Table 2:** Mean Obesity Prevalence by High/Low % Hispanic, % Bachelor’s Degree and % Poverty ^†^.

Category	Mean	*p* value
**% Hispanic X % Bachelor’s Degree**		
High % Hispanic/High % Bachelor’s Degree	24.6	Ref. Cat.
High % Hispanic/Low % Bachelor’s Degree	32.1	0.0000
Low % Hispanic/Low % Bachelor’s Degree	29.0	0.0005
Low % Hispanic/High % Bachelor’s Degree	26.9	0.0957
**% Hispanic X % Poverty**		
High % Hispanic/High % Poverty	29.8	Ref. Cat.
High % Hispanic/Low % Poverty	25.2	0.0023
Low % Hispanic/Low % Poverty	26.7	0.0270
Low % Hispanic/High % Poverty	29.9	0.9261

^†^ High is at or above the mean county level proportion. For example “high” for % Hispanic would be at or above 25.1%, while “low” would be below 25.1% county level % Hispanic.

**Figure 1:**
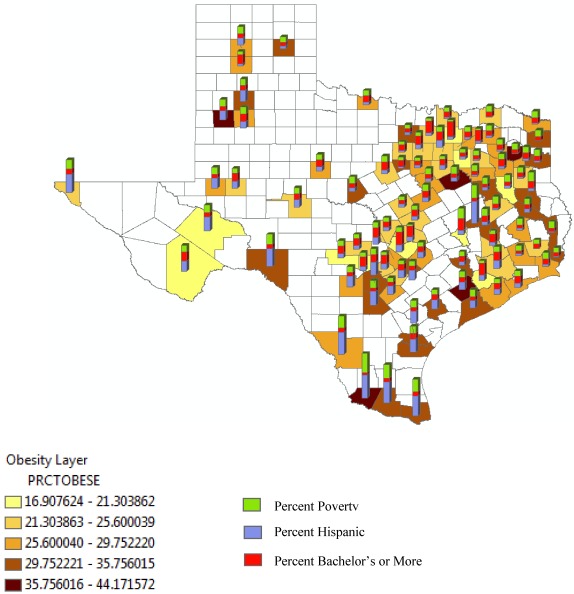
Texas Counties Showing the Association of Percentage of Obesity, Hispanic Population, Poverty, and Graduation with Bachelors or More.

Figure 1 shows a map of the 91 counties by % obese, % Hispanic, % with bachelor’s degree or more and percent poverty. Counties that tend to have a large proportion of obese, also tend to have a high % Hispanics, high % poverty or a low percentage with a bachelor’s degree or more. In fact, in extreme South Texas (*i.e.*, Brownsville region), where there is the highest concentration of Hispanics and those living at or below the poverty line, there is a clustering of counties with percent obese of 29.75 or higher.

Table 3 presents robust regression analysis results for county-level percent obese. In the unadjusted model (Model 1), % Hispanic is significantly associated with percent obesity, so that for every 5.9 increase in % Hispanic, the county-level proportion obese is increased by 1% (*p* = 0.014). Model 2 includes a quadratic equation for % Hispanic. The significant *p* value (*p* = 0.028) of the quadratic term suggests that the relationship between % Hispanic and obesity is not linear but likely to be more of an upside down J-shape. The shape of this relationship is indicated by the positive value of the beta coefficient for the quadratic term and the negative value of the % Hispanic beta coefficient in Model 2. Model 3 includes % with a bachelor’s degree or more without the other covariates. While the relationship tends to be negative, only the 4th Quartile of % bachelor’s degree or more is associated with a significantly lower county prevalence of obesity (β = −6.32, *p* = 0.000). Model 4 includes % poverty. In this model all quartiles are significantly higher than the lowest, suggesting that increased county poverty levels are associated with a significant increase in county obesity prevalence. In the full model, Model 5, the effects for both % Hispanic and % below the poverty line are accounted for and no longer significant. In order to further evaluate this relationship, two addition models are presented in Table 3. The further analysis in Models 6 and 7 demonstrate that the effect of % Hispanic and % poverty are both attenuated by % bachelor’s degree or more.

In a stratified by high/low % poverty and % bachelor’s degree regression model, shown in Table 4 there was a significant relationship between % Hispanic and obesity in counties where more than 50% of the population had a bachelor’s degree (β = −0.193, *p* = 0.007) and in counties where less than 50% of the population lived below the poverty line (β = −0.625, *p* = 0.024). However, the relationship between % Hispanic and % obesity in the counties with less than 50% poverty is non-linear as indicated by the significant % Hispanic quadratic term (β = 0.015, *p* = 0.017). 

**Table 3:** Robust regression analysis results for county-level percent obesity ^†^.

	Model 1	Model 2	Model 3	Model 4	Model 5	Model 6	Model 7
**% Hispanic**	0.059 (0.014)	−0.108 (0.181)			−0.016 (0.826)	−0.106 (0.182)	
% Hispanic ^2 ^*		0.002 (0.028)			0.0004 (0.586)	0.002 (0.075)	
**% Bachelor Degree or more**							
2nd Quartile			1.31 (0.300)		1.10 (0.414)		1.54 (0.221)
3rd Quartile			−1.62 (0.201)		−0.960 (0.481)		−0.818 (0.526)
4th Quartile			−6.32 (0.000)		−5.13 (0.001)		−5.04 (0.000)
**% Below the Poverty Line**							
2nd Quartile				2.93 (0.041)	1.68 (0.203)	2.97 (0.046)	1.69 (0.188)
3rd Quartile				4.25 (0.003)	2.32 (0.088)	4.08 (0.006)	2.34 (0.074)
4th Quartile				6.04 (0.000)	2.87 (0.082)	4.82 (0.008)	3.57 (0.010)

^†^ β coefficient with *p* value in parentheses; * % Hispanic ^2^ is the quadratic term for % Hispanic; Model 1 = % Hispanic; Model 2 = Model 1 + % Hispanic quadratic term; Model 3 = % Bachelors degree only; Model 4 = % Below the Poverty Line only; Model 5 = Model 2 + Model 3 + Model 4; Model 6 = Model 2 + Model 4; Model 7 = Model 3 + Model 4.

**Table 4:** Robust regression analysis results for county-level percent obesity stratified by high/low percent poverty and percent with bachelor’s degree ^†^.

	% Bachelor’s Degree				% Poverty			
	High		Low		High		Low	
	Model 1	Model 2	Model 1	Model 2	Model 1	Model 2	Model 1	Model 2
**% Hispanic**	−0.193 (0.007)	−0.320 (0.175)	0.043 (0.127)	−0.001 (0.917)	0.037 (0.221)	−0.124 (0.267)	0.010 (0.888)	−0.625 (0.024)
% Hispanic ^2 ^*		0.003 (0.582)		0.001 (0.582)		0.002 (0.126)		0.015 (0.017)

^†^ β coefficient with p-value in parentheses; * % Hispanic ^2^ is the quadratic term for % Hispanic; Model 1 = % Hispanic; Model 2 = Model 1 + % Hispanic quadratic term.

**Figure 2:**
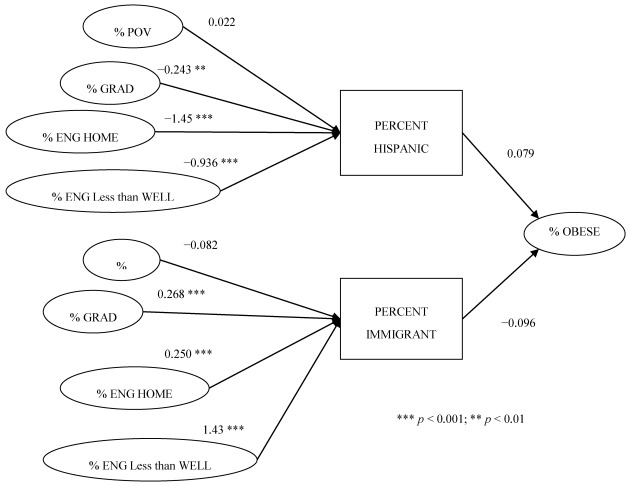
Path analysis for county-level obesity, % Hispanic, % immigrant and potential explanatory variables.

Figure 2 presents the results of path analysis for % Hispanic and % immigrant on % obese. Percent Hispanic has a direct increased association with percent county level obesity (β = 0.079). However, % bachelor’s degree, % who speak English in the home and % Hispanics who speak English less than well all exert significant negative influence of this relationship. For example as the analyses in Table 3 and Table 4 demonstrated, % Hispanic is associated with a decreased prevalence of obesity in counties where 50% or more of its population has a bachelor’s degree. This relationship is shown in the path analysis whereas the coefficient for % Hispanic is (β = 0.079) and the coefficient for % with a bachelor’s degree is β = −0.243. When combined there is a negative effect overall on % obese (*i.e.*, 0.079 + −0.243 = −0.164). The analysis for % immigrant is shown on the bottom portion of the diagram in Figure 1. The direct relationship is negative, however taking into consideration the indirect effects of % Bachelor’s degree, % speak English in the Home, and % speak English less than well the relationship becomes positive at a county level. For example, when combining the effect of % who speak English in the home with percent immigrant the combined effect is 0.154 (−0.096 + 0.250 = 0.154).

**Figure 3:**
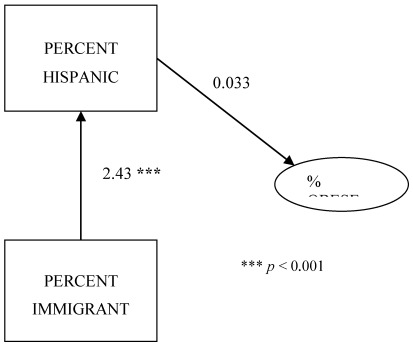
Path analysis for county level obesity, % Hispanic, and % immigrant.

Figure 3 displays the path analysis results for % Hispanic with % immigrant. What this analysis shows, just as in Figure 2, the relationship between % Hispanic and % obese is positive and an increase in % Hispanic corresponds to an increase in % obese at a county level. However, the indirect effect of % immigrant increases the overall combined effect to 2.463. This indicates the combined effect of % Hispanic and % immigrant is associated with greater, not lower, % obese at a county level in Texas.

## 4. Discussion

Past research suggests that Hispanic ethnic concentration can have varying effects on health and mortality outcomes [10,11,12]. Some evidence suggests that it may be protective from early mortality, some forms of cancer, disability, depression and cognitive decline in Mexican Americans. Nevertheless, research also suggests that Hispanic ethnic concentration is a risk factor for late-stage cancer diagnosis, diabetes, and obesity [14,26,27]. We expected that the association of Hispanic ethnic concentration and county-level obesity varies by socioeconomic environment. The findings from this support our expectations in a number of ways. First, we observed differentials in the relationship between % Hispanic and % obese by county level educational attainment. A high proportion of residents with a bachelor’s degree or more was associated with a low prevalence of county-level obesity. Counties with both high % Hispanic and high % with bachelor’s degrees or more had the lowest prevalence of obesity. At the same time, highest prevalence was in high % Hispanic/ low % with bachelor’s degrees. The regression analysis shed light on this association by demonstrating that % bachelor’s degree in a county has greater explanatory effect on the relationship between % Hispanic and % obese than does percent poverty. Still, when stratifying the analysis by high/low % bachelor’s degree and % poverty, we find that % Hispanic is only significantly negative associated with obesity prevalence in communities that more than 50% of the population has a bachelor’s degree and less than 50% of the population live in poverty. Findings that would indicate obesity rates in Hispanic communities are affected by differential socioeconomic conditions in the local environment.

Do *et al*. [14] found that % Hispanic was associated with a higher prevalence of obesity in individuals in the NHANES study. Findings from our study were mixed in their consistency with the results of Do *et al*.’s study. First, % Hispanic was associated with lower county level obesity prevalence in counties where less than 50% of the population were living in poverty or more than 50% of the population had a bachelor’s degree. Second, this study revealed that counties that had 50% or more of the population below the poverty line, there were no differences in Hispanic ethnic composition, but the relationship was positive. The Do *et al*. study used county-level characteristics to predict individual level outcomes, which is different that looking at the county-level as the unit of analysis for the outcome, therefore could explain the differentials in findings. Additionally, the Do *et al*. study did not compare by different socioeconomic levels. 

The relationship between Hispanic ethnic concentration and county-level obesity could also be the result of the cultural tendencies a community overall. To explore this possibility further we conducted a path analysis using % immigrant, % who speak English in the home, and % Hispanics who speak English less than well. Our findings yielded an interesting association that may provide insight into health outcomes associated with obesity in the Southwest [15]. While % Hispanic had a positive effect on % obese, % immigrant had a negative direct effect. When taking into consideration other factors associated with both at a county level, such as % who speak English in the home, the relationships reversed in direction. Therefore, while immigrant concentration may serve as a protective factor potentially for the prevalence of obesity in a county, it depends on other related factors. From similar future analyses, we can further appreciate the complexity of this relationship and looking at multiple factors associated with the cultural environment may provide insight into the pathways at a community or environmental level responsible for inconsistencies in Hispanic disease and comorbidity prevalence’s across the nation. 

At the individual level, in Mexican Americans, greater acculturation to the U.S. mainstream has been associated with lower fruit and vegetable intake [28] and immigrants tend to have greater perceived control over their dietary intake [29]. These differences in behaviors by level of acculturation may explain the differences we observed in the path analysis. Immigrant enclaves in New York City, where there is greater linguistic isolation (*i.e.*, households of respondents where the primary language is other than English, however the dominant language of the surrounding neighborhood is English), have been observed as being associated with healthier eating habits [30]. Additionally, while proportion immigrant was not associated with Body Mass Index (BMI), in New York City, linguistic isolation was [30]. In the path analysis aspect of our study, while we found opposite effects of % Hispanic and % Immigrant, these effects were indirectly influenced by language use and educational attainment. Moreover, the influence of proportion of immigrants in a county contributed to a greater positive association of % Hispanic with county obesity prevalence. A similar finding was observed in Utah using driver’s license data linked to census tract % Hispanic (Latino) and % immigrant [31]. While immigrant concentration was associated with a reduced risk of obesity in the state of Utah, % immigrant amplified this effect. Although the New York City and Utah studies are conducted at different ecological levels compared to our study, they all provide much needed information about the diversity of environments Hispanics live in and how this may exert inconsistencies from one level and one region to the next. In immigrant enclaves, traditions maintained by its inhabitants may be protective from the negative effects of poverty, since immigrant enclaves are often located in the poorest neighborhoods or areas [32].

In the current study, the relationship between Hispanic ethnic concentration was associated with a higher prevalence of county-level obesity in communities with lower educational attainment and greater poverty. While most Hispanic communities in Texas are comprised of primarily U.S. born Mexican Americans, bilingualism is high, whereas Hispanics are fluent in both English and Spanish [22]. Results from our study suggest that the health benefits of living in Hispanic counties may be most beneficial in places with higher educational attainment (e.g., where residents have more knowledge and awareness of both healthy behaviors and the adverse consequences of an unhealthy lifestyle).

Previous national and international studies that have evaluated the impact of socioeconomic environment on obesity have used just income [33] or constructed artificial indices to evaluate risk. This study examined the effects of poverty and education separately and, as a result, found differential effects on Hispanic ethnic concentration and county-level obesity prevalence. Our findings on county-level educational attainment explained the effect of Hispanic ethnic concentration and accounted for a large portion of the effect of poverty. Poverty did not have as strong an effect on the relationship between % Hispanic and % obese as did education in this study. In fact, contrary to what was observed with % with a bachelor’s degree or more, prevalence of obesity did not vary significantly between poor counties that were either high % Hispanic or low % Hispanic. While poverty was significant in the regression model that did not include education, the inclusion of % bachelor’s degree in the final model accounted these effects. Results suggest that poverty may influence access to income-based resources that promote good health and healthy habits [34,35], alternatively it may be that the effect of socioeconomic disadvantage is more complex than income alone and requires separate analysis for education and income.

Relatively few studies have looked at the relationship between obesity and educational attainment at a population level. A study in Cairo, Egypt, found that average neighborhood educational level was associated with BMI [36]. At the individual level, education has been found to be associated with eating behaviors and levels of physical activity [37]. The educational level of a population or community may encourage healthier living through the demand for healthier places to eat, gyms, and outdoor recreational areas and/or greater support for health living policies [38]. This topic deserves further research attention because educational attainment at county-, state- and even country-levels has been associated with many health and mortality outcomes. Improving health education in obesity-prone communities may be an effective, cost efficient way to reverse the obesity epidemic in the U.S.

## 5. Conclusions

Like all studies this one has both strengths and weaknesses. The major limitations include the fact that we were able to include 91 of 254 counties, which may limit the extent we can generalize findings to the entire State of Texas. Larger-scale studies that include other states and a larger number of counties should be considered. We also recognize that the level of data aggregation at a county-level may not be the ideal spatial scale to perform such an analysis, given the risk of ecological fallacy. Yet despite these and other limitations, this study provides insight on an important subject–the effect of Hispanic ethnic concentration and socioeconomic environment on obesity prevalence at a population level.

Most previous studies have looked at obesity in terms of individual behaviors or demographic characteristics and, subsequently, prevention efforts understandably focused on modifying individual behaviors. But these efforts, while well intentioned, have often ignored the community environment in which behaviors that are the focus of change-inducing interventions are occurring. Moreover, many past intervention efforts have been short-lived, leaving people living in the same obesogenic environment as before. Our results suggest that promoting and improving education in populations at risk, perhaps including training on healthful living, may serve as an effective means of curbing current obesity trends and associated health problems in Hispanic and possibly other ethnic communities [39].

## Practical Implications

Obesity is an international public health concern. Understanding risk factors at a community level will help create strategies for implementation of public health policy to curb existing obesity trends. Investing in education has many benefits to a society, one of which may be contributing to the reduction of increased incidence of obesity.
